# Arabidopsis SIGMA FACTOR BINDING PROTEIN1 (SIB1) and SIB2 inhibit WRKY75 function in abscisic acid-mediated leaf senescence and seed germination

**DOI:** 10.1093/jxb/erab391

**Published:** 2021-08-26

**Authors:** Haiyan Zhang, Liping Zhang, Yunrui Ji, Yifen Jing, Lanxin Li, Yanli Chen, Ruling Wang, Huimin Zhang, Diqiu Yu, Ligang Chen

**Affiliations:** 1 CAS Key Laboratory of Tropical Plant Resources and Sustainable Use, Xishuangbanna Tropical Botanical Garden, Chinese Academy of Sciences, Mengla, Yunnan 666303, China; 2 Center of Economic Botany, Core Botanical Gardens, Chinese Academy of Sciences, Menglun, Mengla, Yunnan 666303, China; 3 Key Laboratory for Plant Diversity and Biogeography of East Asia, Kunming Institute of Botany, Chinese Academy of Sciences, Kunming, Yunnan 650201, China; 4 University of Chinese Academy of Sciences, Beijing 100049, China; 5 State Key Laboratory for Conservation and Utilization of Bio-Resources in Yunnan, Yunnan University, Kunming 650091, China; 6 University of Birmingham, UK

**Keywords:** Abscisic acid, GOLDEN 2-LIKE1/2, leaf senescence, seed germination, SIGMA FACTOR BINDING PROTEIN, WRKY75

## Abstract

The plant-specific VQ gene family participates in diverse physiological processes but little information is available on their role in leaf senescence. Here, we show that the VQ motif-containing proteins, Arabidopsis SIGMA FACTOR BINDING PROTEIN1 (SIB1) and SIB2 are negative regulators of abscisic acid (ABA)-mediated leaf senescence. Loss of *SIB1* and *SIB2* function resulted in increased sensitivity of ABA-induced leaf senescence. In contrast, overexpression of *SIB1* significantly delayed this process. Moreover, biochemical studies revealed that SIBs interact with WRKY75 transcription factor. Loss of *WRKY75* function decreased sensitivity to ABA-induced leaf senescence, while overexpression of *WRKY75* significantly accelerated this process. Chromatin immunoprecipitation assays revealed that WRKY75 directly binds to the promoters of *GOLDEN 2-LIKE1*(*GLK1*) and *GLK2*, to repress their expression. SIBs repress the transcriptional function of WRKY75 and negatively regulate ABA-induced leaf senescence in a *WRKY75*-dependent manner. In contrast, *WRKY75* positively modulates ABA-mediated leaf senescence in a *GLK*-dependent manner. In addition, *SIB*s inhibit *WRKY75* function in ABA-mediated seed germination. These results demonstrate that SIBs can form a complex with WRKY75 to regulate ABA-mediated leaf senescence and seed germination.

## Introduction

Leaf senescence constitutes the last stage of plant development and is an evolutionarily selected developmental process that is controlled by a highly regulated genetic network. Numerous studies have demonstrated that leaf senescence is critical for plant growth and also increases reproductive success and fitness, because it enables the relocation of mobilizable nutrient and energy from aging leaves to reproducing seeds (Lim *et al.*, 2007). Thus, plant senescence represents an important adaptive mechanism that plants use to increase their survival and fitness in their given ecological niches. Senescence is initiated in an age-dependent manner and is also triggered by environmental signals and various phytohormones, including abscisic acid (ABA), jasmonic acid (JA), salicylic acid (SA), ethylene (ET), gibberellin (GA) and brassinosteroids, but inhibited by cytokinins and auxin (Lim *et al.*,2007; Chen *et al.*, 2017).

ABA is known as an important regulator that mediates both plant growth and response to diverse environmental stresses (Fujii and Zhu, 2009). It coordinates a sophisticated gene regulatory network to enable plants to respond properly to both developmental and environmental signals (Hubbard *et al.*, 2010; Chen *et al.*, 2020). Three protein classes have been identified as major components that form a central signaling module to govern ABA signal transduction (Hubbard *et al.*, 2010). These components are composed of the membrane-localized receptors PYRABACTIN RESISTANCE (PYR)/PYR1-LIKE (PYL)/REGULATORY COMPONENT OF ABSCISIC ACID RECEPTOR (RCAR), protein phosphatases type 2Cs (PP2Cs) and SNF1-related kinases 2 (SnRK2s). Under normal conditions, PP2Cs interact with, and dephosphorylate SnRK2s, causing their reduction of catalytic activities (Umezawa *et al.*, 2009; Vlad*et al*., 2009; Dupeux *et al.*, 2011). A raise in ABA concentrations results in PYR/PYL/RCAR receptor-mediated repression of PP2C activity, leading to the activation of SnRK2s, which then phosphorylate downstream transcription factors (TFs) and ultimately activate ABA signaling (Umezawa *et al.*, 2009; Vlad *et al.*, 2009; Dupeux *et al.*, 2011). Interestingly, one recent study revealed that the GARP family of transcription factors GOLDEN 2-LIKE1 (GLK1) and GLK2 can form a transcription module with WRKY40 to suppress *ABSCISIC ACID INSENSITIVE 5* (*ABI5*) expression upon activation via the ABA signaling components PYL/PYRs-PP2Cs-SnRKs, to finally modulate the ABA response (Ahmad *et al.*, 2019). Although numerous studies have demonstrated that ABA plays an important role in diverse physiological processes, such as leaf senescence and seed germination, the underlying mechanisms involved remain to be further investigated.

Recently, a class of plant-specific transcriptional regulators with a short conserved VQ motif (FxxhVQxhTG) was identified and designated as VQ proteins (Jing and Lin, 2015; Yuan *et al.*, 2021). Several studies have demonstrated that the VQ motif has a great impact on the actions or functions of VQ proteins. For instance, the VQ motif has been demonstrated to be essential for both protein–protein interactions and protein sub-cellular localization, and modification of the VQ motif can also change the transcriptional activities of VQ proteins (Jing and Lin, 2015; Jiang and Yu, 2016; Yuan *et al.*, 2021). As a family of transcriptional regulators, VQ proteins often work in concert with their interacting partners to fine-tune the complex regulatory networks that mediate plant growth and stress responses. For example, the structurally related VQ proteins, SIGMA FACTOR BINDING PROTEIN1 (SIB1) and SIB2, function as transcriptional activators of WRKY33 to modulate plant defense against *Botrytis cinerea* (Lai *et al.*, 2011). In contrast, VQ9 functions with WRKY8 to modulate the plant salt stress response (Hu *et al.*, 2013). The VQ protein IKU1 (VQ14) interacts with MINI3 (WRKY10) to regulate endosperm development and seed size (Wang *et al.*, 2010). Besides WRKY transcription factors, VQ proteins also interact with their other interacting partners, such as PHYTOCHROME INTERACTING FACTOR 4 (PIF4), ABI5, mitogen-activated protein kinases (MAPKs), and RING-type E3 ubiquitin ligase, to coordinate diverse physiological processes (Li *et al.*, 2014; Pecher *et al.*, 2014; Pan *et al.*, 2018; Ali *et al.*, 2019). Although several VQ proteins have been functionally characterized, the biological roles of specific VQ proteins under given conditions are largely unknown. Until now, there has been no report about their involvement in leaf senescence, and thus it is worthwhile to investigate their biological significance in this process.

As a class of important interacting partners of VQ proteins, the WRKY transcription factor family has been shown to form integral parts of the complex signaling networks to regulate both plant growth and stress responses (Rushton *et al.*, 2010; Chen *et al.*, 2012;^2017^; Guo *et al.*, 2017; Zhang *et al.*, 2018). Recent studies have provided evidence to show that WRKY proteins often interact with important proteins of various phytohormone signaling pathways to regulate diverse physiological processes. For example, WRKY57 can form a complex with both repressors of the JA and auxin signaling pathways, including JASMONATE ZIM-DOMAIN4/8 (JAZ4/8) and the AUX/IAA protein IAA29, and thus function as a node of convergence for JA- and auxin-mediated signaling pathways in JA-induced leaf senescence (Jiang *et al.*, 2014). WRKY45 physically associates with a repressor of the GA signaling pathway, such as the DELLA protein RGA-LIKE1 (RGL1), to positively regulate leaf senescence (Chen *et al.*, 2017). In addition, WRKY12/13 and WKKY75 also participate in GA-mediated floral initiation via interaction with DELLAs (Li *et al.*, 2016; Zhang *et al.*, 2018). Furthermore, WRKY proteins also act as critical components of various phytohormone-mediated signaling pathways to mediate various plant processes. For instance, the structurally related WRKY proteins, including WRKY18/40/60, directly bind to the promoter of several ABA-responsive genes, such as *ABI4* and *ABI5*, to regulate ABA-mediated seed germination(Shang *et al.*, 2010).Similarly, WRKY75 can directly associate with the promoters of *SA INDUCTION-DEFICIENT2* (*SID2*) and *ETHYLENE RESPONSIVE FACTOR 59* (*ORA59*), to regulate SA-promoted leaf senescence. and JA-mediated plant defense to necrotrophic fungal pathogens, respectively (Guo *et al.*, 2017; Chen *et al.*, 2021). However, it is still unclear whether WRKY proteins can function together with their interacting partners, such as VQ proteins, to participate in modulation of ABA-mediated leaf senescence or seed germination.

In this study, in order to explore the possible roles of VQ-WRKY complexes in ABA-mediated leaf senescence or seed germination, we used both molecular and genetic approaches to investigate the physiological effects between SIBs and WRKY75 in these processes. Our results indicated that SIBs function as repressors of WRKY75, and regulate expression of *GLK*s in ABA-mediated leaf senescence and seed germination.

## Materials and methods

### Plant growth conditions and materials

Plants used in this study were derived from Arabidopsis Col-0 ecotype. Seeds were surface sterilized with 20% bleach for 15 min and sown on half-strength Murashige and Skoog (MS) media for 3 d at 4 °C. Plants were transferred to soil 7 d after germination and were grown in a greenhouse at 22 °C under a 16h light/8h dark photoperiod. *N. benthamiana* were grown in a green house at 25 °C under a 16 h light/8 h dark photoperiod. The *wrky75-1*(SALK_101367), *wrky75-25*, *sib1-4*(SM_3.30596), *sib2-1* (SM_3.16236), *WRKY75:YFP-WRKY75:3’-WRKY75*, and *35S:WRKY75-L3* have been described in previous studies(Rishmawi *et al.*, 2014; Zhang *et al.*, 2018; Lv *et al.*, 2019).To generate *SIB1* overexpression transgenic plants, the full-length cDNA of *SIB1* was cloned into the binary vector *pOCA30* in the sense orientation behind a CaMV*35S* promoter. Taq DNA polymerase was purchased from Takara Biotechnology Co. Ltd (Japan), ABA was purchased from Sigma Co. Ltd (USA) and other chemicals were purchased from Shanghai Sangon Biotechnology Co. Ltd (China).

### Expression analysis

ABA was dissolved in 90 µl of ethanol, and water was added to obtain a 10mM stock solution. The ABA stock solution was diluted to 100 µM with distilled water and sprayed onto plants. Water was sprayed onto plants as a control. Total RNA was extracted using Trizol reagent (Invitrogen, USA) from leaves of different ages, or leaves treated with or without 100 µM ABA. About 1 μg of DNase-treated RNA was used for complementary DNA (cDNA) synthesis using M-MLV reverse transcriptase (TaKaRa, Japan), followed by PCR on a Roche LightCycler 480 real-time PCR machine using SYBR Premix Ex Taq™ II (Roche, Mannheim, Germany). *ACTIN2* (AT3G18780) and *UBQ5* (AT3G62250) were used as internal controls in quantitative RT–PCR. Analysis was conducted following the minimum information for publication of quantitative Real-Time PCR experiments guidelines (Bustin *et al.*, 2009). The gene-specific primers used for qRT-PCR are listed in Supplementary Table S1.

### Assays of ABA-induced senescence

The fifth and sixth rosette leaves from 4-week-old plants were placed onto Petri dishes filled with distilled water supplemented with or without 100 µM ABA, and then the plates were kept under weak light (20 µmolm^-2^s^-1^photosynthetic photon flux density)for 0, 4 or 5 d at 22 °C. Three-week-old plants grown in soil were sprayed with or without 100 µM ABA and placed under weak light (20 µmolm^-2^s^-1^ photosynthetic photon flux density) for 3 dat 22 °C.

Chlorophyll of detached leaves was extracted with 80% acetone, according to Lichtenthaler (1987). Cell death rate was detected by Trypan blue staining; the leaves of indicated genotypes treated with or without 100 µM ABA were soaked in 0.05% Trypan blue solution, kept at80 °C for 2 min, and then cleared with chloral hydrate.

### Measurement of germination and greening rates

Seeds were stratified for 3 d at 4 °C. Germination was determined based on the appearance of the embryonic axis (i.e. radicle protrusion), as observed under a microscope (Olympus, Japan). Seedling greening was determined based on the appearance of green cotyledons in a seedling. To measure the ABA sensitivity of germination and greening, seeds were plated on half-strength MS supplemented with different concentrations of ABA(0, 0.5, 0.75, or 1µM). Three independent experiments were conducted, and similar results were obtained.

### Yeast two-hybrid screening and confirmation

The coding sequences of full-length *WRKY75* (from 35S:WRKY75 construct; Zhang *et al.*, 2018 ) and its derivatives were cloned into bait vector pGBKT7 (Clontech, USA), which was transformed into the yeast strain Y2H Gold (Clontech, USA). Two-hybrid screening was performed according to the mating protocol described in the Clontech Matchmaker TM Gold Yeast Two-Hybrid user manual. To confirm protein-protein interactions, the coding sequences of full-length *SIB1*or *SIB2* which were amplified from a senescence-associated cDNA library and their derivatives were fused to the prey vector pGADT7. The primers used for Y2H screening are shown in Supplementary Table S1.

### LUC complementation imaging assays

The full-length *WRKY75* CDS was cloned intopCAMBIA1300*-nLUC*, and the full-length *SIB1* or *SIB2* CDS were cloned into pCAMBIA1300*-cLUC*. All plasmids were introduced into *Agrobacterium tumefaciens* strainEHA105,and then infiltration of *N. benthamiana* leaves was performed as described in Chen *et al*. (2008). Infected leaves were analyzed 72 h after infiltration under a low-light cooled CCD imaging apparatus (Tanon-5200, China) to acquire the LUC images. Before luminescence detection, the leaves were sprayed with 0.5 mM fluorescein and kept in the dark for 5 min. The primers used for LUC complementation imaging assays (LCI) are shown in Supplementary Table S1.

### Pull-down and electrophoretic mobility shift assay (EMSA)

The full-length *SIB1* and *WRKY75* CDS were cloned intopGEX-4T-1 (Zhang *et al.*, 2018), and the full-length *WRKY75* CDS was cloned into pET-28a(+). All plasmids were introduced into *Escherichia coli* BL21 cells, and Glutathione S-transferase (GST), GST-SIB1, and His-WRKY75 protein expression was induced by 0.5 mM isopropyl-b-thiogalactopyranoside for 24 h at 16 °C. Soluble GST and GST-SIB1 were extracted and immobilized to glutathione beads (Thermo Fisher Scientific, USA). His-WRKY75 fusion protein from *E. coli* cell lysate was co-incubated with the immobilized GST and GST–SIB1 fusion proteins for 4 h at 4 °C. Proteins were eluted with elution buffer, and then western blot was used to determine the interaction between WRKY75 and SIB1. The purified GST-WRKY75 protein was confirmed by SDS-PAGE and used for EMSA. EMSA was performed using a Chemiluminescent EMSA Kit (Beyotime, China).The probes were synthesized and labeled with biotin at the Beijing Genomics Institute.

### GUS staining and determination of YFP fluorescence

GUS staining was performed as described by Chen *et al*.(2021). Seeds or 10-day-old seedlings of *WRKY75p*:*GUS* transgenic plants floated on water or 100 µM ABA were used as materials for GUS staining. Chlorophyll was removed using several changes of 70% (v/v) ethanol, and the tissues were subsequently photographed. Furthermore, roots of *WRKY75:YFP-WRKY75:3’-WRKY75* plants were treated with water or ABA, and then YFP fluorescence was observed under a confocal laser scanning microscope (Olympus, Japan; Rishmawi *et al.*, 2014).

### Chromatin immunoprecipitation assays

Two-week-old and ABA-pre-treated seedlings of Col-0and*Myc-WRKY75* were harvested for chromatin immunoprecipitation(ChIP) assays, as described previously (Saleh *et al.*, 2008).The Myc antibody was used to immunoprecipitate the protein-DNA complex, and the precipitated DNA was purified using a PCR purification kit for qRT–PCR analysis. The ChIP experiments were performed three times. Chromatin precipitated without antibody was used as the negative control, whereas the isolated chromatin before precipitation was used as the input control. ChIP results are presented as a percentage of input DNA. The primers used for qRT–PCR amplification of different promoters are listed in Supplementary Table S1.

### Transcriptional activity assays

The native promoter (about 2.6kb)of *GLK1* was inserted into pGWB35 to generate a *GLK1p:LUC* reporter construct using Gateway technology (Invitrogen, USA).The reporter plasmid and the constructs containing *35S:WRKY75*, *35S:SIB1* and *35S:SIB2* were transformed into *A. tumefaciens* strain EHA105. The strains were incubated overnight in Luria-Bertani medium and resuspended in infiltration buffer (10 mM MES, 0.2 mM acetosyringone, and 10 mM MgCl_2_) at an optical density at OD_600_=1. Equal amounts of different combined bacterial suspensions were infiltrated into the 5-week-old *N. benthamiana* leaves using a needleless syringe. After infiltration, the LUC images were acquired under a low-light cooled CCD imaging apparatus (Tanon-5200, China). Experiments were performed with three independent biological replicates, and generated similar results.

### Statistical analysis

Statistical analysis between samples were performed by Student’s *t*-test or analysis of variance (ANOVA). Sample differences were considered to be statistically significant (indicated with * or different letters) if *P*<0.05.

## Results

### *SIB*s negatively modulate ABA-mediated leaf senescence and seed germination

**Fig. 1.**
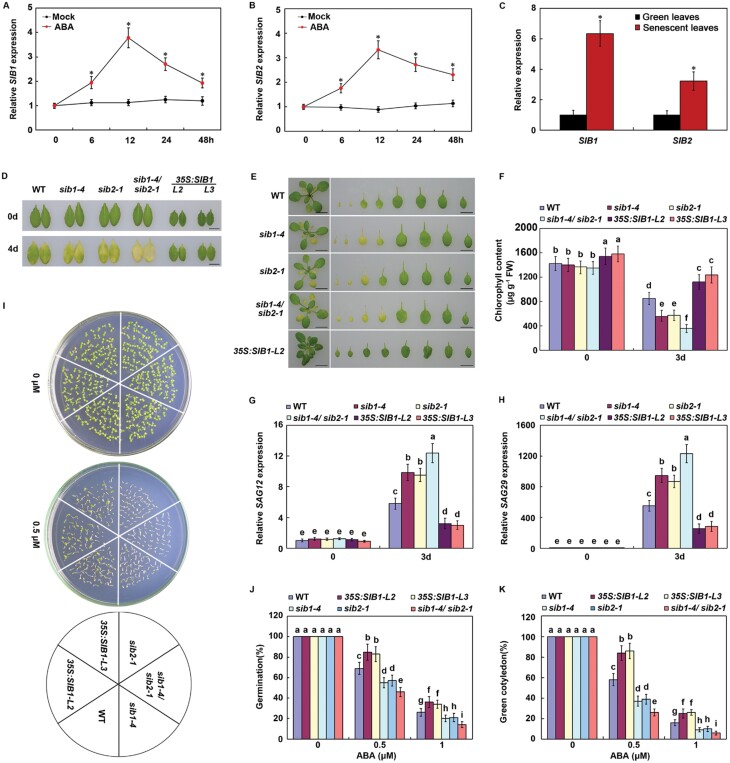
*SIB1/2* negatively modulate ABA-induced leaf senescence and seed germination.(A, B). qRT–PCR analysis of *SIB1* and *SIB2* transcript levels in 4-week-old WT leaves upon 100 µM ABA treatment. (C) qRT–PCR analysis of *SIB1* and *SIB2* transcript levels in green and senescent leaves. For A-C, transcript levels of *SIB1* and *SIB2* in untreated or non-senescent leaves were arbitrarily set to 1. *ACTIN2* and *UBQ5* were used as internal controls. Error bars represent ±SD from three independent biological replicates. *P<0.05, Student’s *t*-test compared with mock or green leaves. (D) Senescence phenotypes of the 4-week-old detached leaves of the indicated genotypes treated with or without 100 µM ABA for 4 d. Scale bar =1 cm. (E) Senescence phenotypes of the indicated genotypes treated with or without 100 µM ABA for 4 d. Scale bar =1cm. (F) Chlorophyll content in the indicated genotypes treated with or without 100 µM ABA for 3 d. (G,H) qRT–PCR analysis of *SAG12* and *SAG29* expression in the leaves of the indicated genotypes treated with or without 100 µM ABA for 3 d. Transcript levels of *SAG12* or *SAG29* in untreated green leaves were arbitrarily set to 1. *ACTIN2*and *UBQ5* were used as internal controls. (I) Phenotypes of the indicated genotypes grown on half-strength MS medium with 0 or 0.5 µM ABA for 5 d. (J) Germination rates of the indicated genotypes grown on half-strength MS medium with 0, 0.5, or 1 µM ABA for 3 d (K) Cotyledon greening rates of the indicated genotypes grown on half-strength MS medium with 0, 0.5, or 1 µM ABA for 6 d. For F-H, J, and K, error bars represent ±SD from three independent biological replicates. Bars with different letters are significantly different from each other (ANOVA; *P*<0.05).

*SIB1* and *SIB2* were previously found to function as important regulators in JA-mediated defense against necrotrophic pathogens and SA-primed cell death (Lai *et al.*, 2011; Li *et al.*, 2020). To investigate the possible involvement of *SIB*s in ABA-mediated responses, we first examined the inducibility and temporal kinetics of both *SIB1* and *SIB2* expression upon ABA treatment. As shown in Fig. 1A, B, expression of both *SIB1* and *SIB2* was induced by ABA. Further expression analysis showed that *SIB1* and *SIB2* also have strong expression in senescent leaves (Fig. 1C). Thus, these results imply that *SIB1* and *SIB2* may play a role in ABA-induced leaf senescence. Next, their single and double mutants were used to investigate their function in ABA-induced leaf senescence (Lai *et al.*, 2011; Lv *et al.*, 2019; Li *et al.*, 2020). Both the detached leaves and the whole plants of the wild type, *sib*s single and double mutants, or *SIB1* overexpression lines (Supplementary Fig. S1), were used for ABA-induced leaf senescence assays. Upon ABA treatment, the *sib1* and *sib2* single mutants showed more serious yellowing, which is a typical characteristic of leaf senescence, when compared with wildtype (WT; Fig. 1D, E). Interestingly, the *sib1/sib2* double mutant further showed more serious yellowing than the single mutants, implying that *SIB*s may function redundantly in ABA-induced leaf senescence (Fig. 1D, E). Consistent with these findings, chlorophyll content was lower, and expression of representative *SENESCENCE ASSOCIATED GENES* (*SAG*s; e.g. *SAG12* and *SAG29*) was stronger in *sib* mutants than in WT plants (Fig. 1F–H). In contrast, *35S:SIB1* transgenic plants (*35S:SIB1-L2* and*35S:SIB1-L3*) showed remarkably delayed leaf senescence upon ABA treatment, accompanied with higher chlorophyll content, but lower *SAG* expression, compared with WT (Fig. 1D–H; Supplementary Fig. S1). Together, these observations suggest that *SIB*s negatively modulate ABA-induced leaf senescence.

Because both *SIB1* and *SIB2* are induced by ABA, we hypothesized that they may also participate in other ABA-mediated responses, such as seed germination. To confirm our hypothesis, we compared the phenotypes of the wild type, *sib*s single and double mutants, and *SIB1* overexpression lines, in response to ABA during seed germination. As shown in Fig. 1I–K, there were no great differences in phenotypes among WT, *sib*s single and double mutants, and *SIB1* overexpression lines, on half-strength MS medium. Subsequently, we further investigated the phenotype of these seeds after treatment with exogenous ABA. As shown in Fig. 1I–K, the *sib1* and *sib2* single mutant seeds were more sensitive to ABA compared with WT during seed germination and post-germinative growth. Furthermore, the *sib1/sib2* double mutant seeds showed even more sensitivity to ABA compared with their single mutants, implying that *SIB*s also function redundantly in ABA-mediated seed germination and post-germinative growth (Fig. 1I–K). In contrast, the seeds of *35S:SIB1* transgenic plants showed much higher germination and greening cotyledons than the WT (Fig. 1I–K). Thus, our results support the notion that SIBs function as negative regulators in ABA-mediated seed germination and early seedling growth.

### Physical interaction between SIBs and WRKY75

**Fig. 2.**
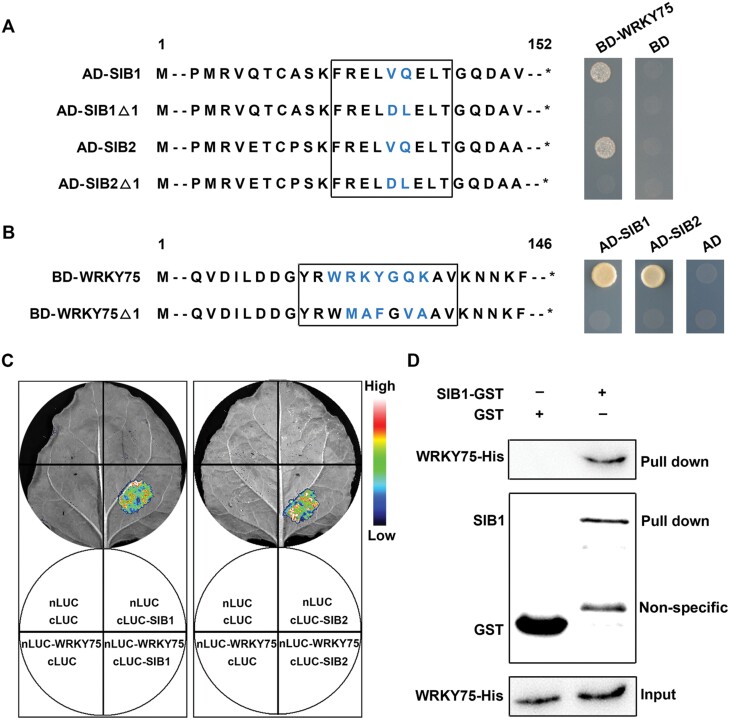
SIB1 and SIB2 physically interact with WRKY75. (A) Yeast-two-hybrid assays. The VQ domains of SIB1 and SIB2 are necessary for their interaction with WRKY75. Sequences of full-length and mutated SIB1 or SIB2 were fused to the pGADT7 activation domain (AD, prey), and sequences of full-length WRKY75 were fused to the pGBKT7 binding domain (BD, bait). Interactions were indicated by the ability of yeast cells to grow on selective media lacking Leu, Trp, His, and Ade. The empty pGBKT7 prey vector was used as a negative control. *Represents stop codon. (B) Yeast-two-hybrid assays. The WRKYG domain of WRKY75 is essential for its interaction with SIB1 and SIB2. Sequences of full-length and mutated WRKY75 were fused to the pGBKT7 binding domain (BD, bait), sequences of full-length SIB1 and SIB2 were fused to the pGADT7 activation domain (AD, prey). Interactions were indicated by the ability of yeast cells to grow on selective media lacking Leu, Trp, His, and Ade. The empty pGADT7 prey vector was used as a negative control. (C) LUC complementation imaging (LCI) assay detecting the interaction between WRKY75 and SIB1 or SIB2. Images of *N. benthamiana* leaves are displayed two days after infiltration. The pseudocolor bar shows the range of luminescence intensity. (D) *In vitro* pull-down assays. Purified GST or SIB1–GST was incubated with the WRKY75–His protein. GST was used as a negative control.

During our screening of potential interaction partners of WRKY75using the yeast two-hybrid system (Y2H), we found that WRKY75 can interact with both SIB1 and SIB2 (Chen *et al.*, 2021). Furthermore, the biological significance of their interaction remains to be determined. Interestingly, WRKY75 has been revealed to participate in several physiological processes through JA, GA, or SA pathways (Guo *et al.*, 2017; Zhang *et al.*, 2018; 2021; Chen *et al.*, 2021). Thus, we speculated that WRKY75 may form a complex with SIBs to co-regulate ABA mediated responses. We further confirmed their interaction using Y2H and luciferase complementation imaging (LCI) assay. As shown in Fig. 2A, B, both SIB1 and SIB2 interact with WRKY75, and the WRKYGQK sequence and VQ-motif are essential for their interaction. For the LCI assay, *cLUC-SIB*s*/nLUC-WRKY75*, *cLUC/nLUC*, *cLUC-SIB*s*/nLUC*, and *cLUC/nLUC-WRKY75* were co-expressed together in *Nicotiana benthamiana* leaves at the same time. As shown in Fig. 2C, a LUC signal was only detected when *cLUC-SIBs/nLUC-WRKY75* were co-injected into the *N.benthamiana* leaves. We also confirmed their interactions by performing *in vitro* pull-down assays. The results of the assay showed that the GST-fused SIB1 was able to retain WRKY75-His, whereas GST alone could not (Fig. 2D). Taken together, these results demonstrate that SIBs physically interact with WRKY75 and they may form a complex to co-regulate ABA-mediated leaf senescence and seed germination.

### WRKY75 positively regulates ABA-mediated leaf senescence and seed germination

**Fig. 3.**
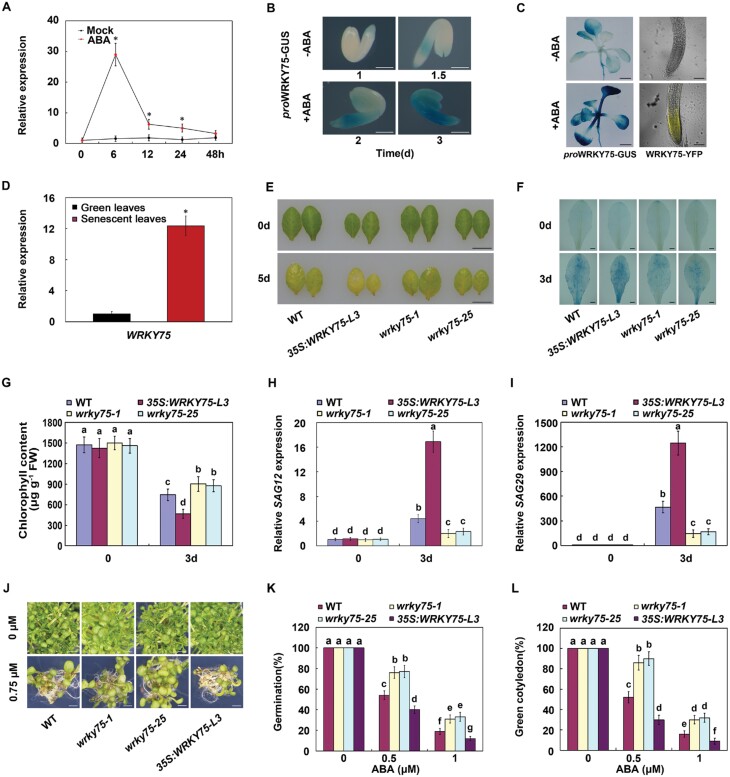
WRKY75 positively regulates ABA-induced leaf senescence and seed germination. (A) qRT–PCR analysis of *WRKY75* transcript levels in 4-week-old WT leaves upon 100 µM ABA treatment . Transcript levels of *WRKY75* in untreated leaves were arbitrarily set to 1. *ACTIN2* and *UBQ5* were used as internal controls. Error bars represent ±SD from three independent biological replicates. *P<0.05, Student’s *t*-test compared with mock treatment. (B) GUS expression in *WRKY75p*:*GUS* transgenic seedlings grown on half-strength MS medium at the indicated times, treated with or without 100 µM ABA. Scale bar =200 µm. (C) Left: GUS staining of 10-d-old *WRKY75p*:*GUS* seedlings treated with or without 100 µM ABA for 2 h. Scale bar=1mm; Right: YFP detection of *WRKY75* in 5-d-old *WRKY75:YFP-WRKY75:3’-WRKY75* seedlings treated with or without ABA for 2 h. Scale bar =100 µm. (D) qRT–PCR analysis of *WRKY75* transcript levels in 24-day-old non-senescent and 40-day-old senescent leaves. Transcript levels of *WRKY75* in non-senescent leaves were arbitrarily set to 1. *ACTIN2*and *UBQ5* were used as internal controls. Error bars represent ±SD from three independent biological replicates.*P<0.05, Student’s *t*-test compared with green leaves. (E)Senescence phenotypes of the 4-week-old leaves of the indicated genotypes treated with or without 100 µM ABA for 4 d. Scale bar =1 cm. (F) Trypan blue staining of the indicated genotypes treated with or without ABA for 3 d. Scale bar =2 mm. (G) Chlorophyll content in the indicated genotypes. (H, I) qRT–PCR analysis of *SAG12* and *SAG29* transcript levels in the indicated genotypes. Transcript levels of *WRKY75* in control WT leaves were arbitrarily set to 1. *ACTIN2* and *UBQ5* were used as internal controls. (J) Phenotypes of the indicated genotypes on half-strength MS medium with 0 or 0.75 µM ABA for 7 d. Scale bar =2 mm. (K) Germination rates of the indicated genotypes on half-strength MS medium with 0, 0.5, or 1 µM ABA treatment for 2 d. (L) Greening rates of the indicated genotypes on half-strength MS medium with 0, 0.5, or 1 µM ABA for 6 d. For G-I, K, and L, error bars represent ±SD from three independent biological replicates. Bars with different letters are significantly different from each other (ANOVA; *P*<0.05).

Since SIBs participate in ABA-mediated leaf senescence and seed germination and interact with WRKY75, we speculated that WRKY75 may also participate in ABA responses through interaction with SIBs. To investigate the possible involvement of *WRKY75* in ABA-mediated responses, we first determined the induced expression of *WRKY75* upon ABA treatment. As shown in Fig. 3A–C, *WRKY75* expression was induced by ABA at both mRNA and protein levels. Combined with its strong expression in senescent leaves (Fig. 3D; Guo *et al.*, 2017; Zhang *et al.*, 2021), we deduced that *WRKY75* may also play a role in ABA-induced leaf senescence. Therefore, the detached leaves of the WT, *wrky75* mutants, or *WRKY75* overexpression plants were used for ABA-induced leaf senescence assays. Upon ABA treatment, the *wrky75* mutants showed delayed leaf senescence compared with WT plants (Fig. 3E). The mutant plants also displayed decreased cell death, higher chlorophyll content and lower expression of *SAG*s than WT plants (Fig. 3F–I). In contrast, *35S:WRKY75* transgenic plants showed accelerated leaf senescence upon ABA treatment, accompanied by enhanced cell death and lower chlorophyll content, but higher *SAG* expression (Fig. 3F–I). Thus, these observations suggest that *WRKY75* positively modulates ABA-induced leaf senescence.

Similarly, we also used seeds of WT, *wrky75* mutants, and *WRKY75* overexpression plants to determine possible participation of *WRKY75* in ABA-mediated seed germination. As shown in Fig. 3J–L, there were no great differences among WT, *wrky75* mutants, and *WRKY75* overexpression plants on half-strength MS medium. We further investigated the phenotype of these seeds after treatment with exogenous ABA. As shown in Fig. 3J–L, the *wrky75* mutant seeds were more insensitive to ABA compared with WT during seed germination and post-germinative growth. In contrast, seeds of the *35S:WRKY75* transgenic plants showed much lower germination and greening cotyledons than the WT (Fig. 3J–L). Taken together, our results support the notion that WRKY75 also functions as a positive regulator in ABA-mediated seed germination and post-germinative growth.

### WRKY75 binds to the promoters of *GLK1* and *GLK2* to inhibit gene expression

WRKY transcription factors often perform their biological functions by specially binding to the W-box (T/CTGACC/T), present in the promoters of their target genes (Eulgem *et al.*, 2000; Ulker and Somssich, 2004). Our results indicate that WRKY75 may play a role in ABA-mediated leaf senescence and seed germination by modulating the expression of both senescence and seed germination-associated genes in the ABA signaling pathway. Interestingly, several putative W-box elements were found to be present in promoters of*GLK1* and *GLK2*. Previous studies have demonstrated that GLKs participate in both ABA-mediated leaf senescence and seed germination processes (Rauf *et al.*, 2013; Ahmad *et al.*, 2019). Thus, we speculated that WRKY75 may directly bind the promoters of *GLK*s to inhibit their expression, and finally modulate ABA responses.

**Fig. 4.**
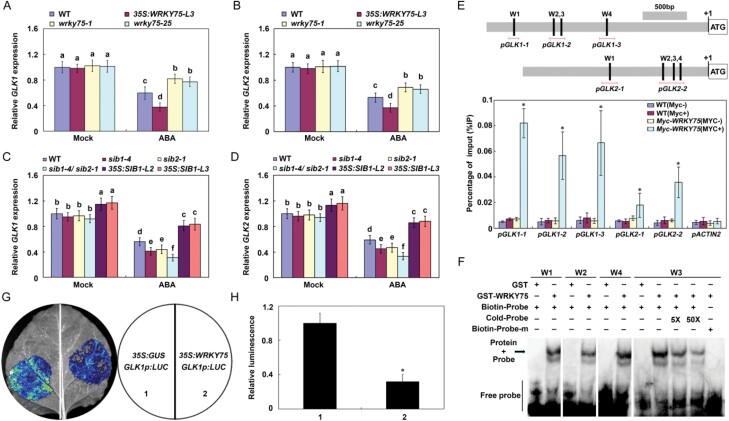
WRKY75 directly represses the expression of *GLK1* and *GLK2.* (A, B).Transcript levels of *GLK1* and *GLK2* in 4-week-old WT, *WRKY75* mutants and overexpressing plants upon 100µM ABA treatment for 12 h. (C, D)Transcript levels of *GLK1* and *GLK2* in the WT, *sib* single or double mutants, and *SIB1* overexpressing plants upon 100µM ABA treatment for 12 h. For A-D, error bars represent ±SD from three independent biological replicates. Bars with different letters are significantly different from each other (ANOVA; *P*<0.05). (E) ChIP-qPCR analysis of the relative binding of WRKY75 to the promoters of *GLK1* and *GLK2*. The promoter structures of both *GLK1* and *GLK2* and fragment used in the ChIP assay. (Upper) W1, W2, etc. denote each W-box, numbered from left to right with sequence sites relative to the ATG start codon. Red lines indicate the sequences detected by ChIP assays. (Bottom) Real-time RT-PCR results showed that WRKY75 binds to the promoters of *GLK1* and *GLK2*. ChIP assays were performed with chromatin prepared from *Myc-WRKY75* plants using an anti-Myc antibody. ChIP results are presented as a percentage of input DNA. Error bars represent ±SD from three independent biological replicates. (F) EMSA of the binding of recombinant WRKY75 proteins to the promoter of *GLK1*. The oligonucleotides (*proGLK1-W1/2/3/4* and *proGLK1-W3-m*) were used as the probes. Mutated probe indicates a single nucleic acid mutation from TGAC to TAAC.GST-WRKY75, biotin-probe, labeled mutated probe, and unlabeled probe at a 5× and 50× molar excess were present (+) or absent (-) in each reaction. (G,H) Transient transcriptional activity assays in *N. benthamiana*. A representative leaf image is shown in (G), and the quantification of corresponding relative luminescence intensities was done in (H) by using *n*=15 independent leaves. Error bars represent ±SD.*P<0.05, Student’s *t*-test compared with control.

To determine whether *GLK*s are direct targets of WRKY75, we then compared their expression in WT, *wrky75* mutants, and *WRKY75* overexpression plants upon ABA treatment. As shown in Fig. 4A, B, the expression of both *GLK1* and *GLK2* was higher in *wrky75* mutants, but was lower in *WRKY75* overexpression plants, compared with those in WT. Since SIBs physically interact with WRKY75 and may function together to regulate ABA-mediated leaf senescence and seed germination, we also compared their expression in WT, *sib* single and double mutants, and *SIB1* overexpression lines, upon ABA treatment. Expression of both *GLK1* and *GLK2* was lower in *sib* mutants, but was higher in *SIB1* overexpression plants compared with those in WT (Fig. 4C, D). Thus, both WRKY75 and SIBs may regulate ABA-mediated responses through *GLK1* and *GLK2*.

To determine whether *GLK*s are direct targets of WRKY75, we then conducted *in vivo* ChIP assays using *35S:Myc-WRKY75* transgenic plants (Zhang *et al.*, 2018). The ChIP-qPCR results revealed that WRKY75 could bind to the promoters of both *GLK1* and *GLK2* via the W-box sequence (Fig. 4E). Furthermore, we also performed EMSAs with the GST-WRKY75 recombinant protein to determine the *in vitro* binding of WRKY75 to the *GLK1* promoter. As shown in Fig. 4F, WRKY75 could bind all the probes containing W-box sequence (W1, W2, W3, and W4). The binding signals decreased after the addition of unlabeled WT competitors. In contrast, the WRKY75 protein did not bind to the mutant probe carrying a mutated W-box (Fig. 4F). The GST protein alone also did not bind to the *GLK1* promoter (Fig. 4F). These data suggest that WRKY75 may directly bind to the promoters of *GLK*s to modulate ABA responses.

Next, to further confirm the direct regulation of WRKY75 on expression of *GLK*s, we also performed transient expression assays in *N. benthamiana* leaves using the *GLK1* promoter (2.6 kb) fused with the *LUC* gene (*GLK1p:LUC*). Effector plasmids were generated that contained either a *WRKY75* or *GUS* gene driven by the Cauliflower mosaic virus (CaMV) *35S* promoter (*35S:WRKY75* and *35S:GUS*). As shown in Fig. 4G, H, co-expression of the *WRKY75* gene with the reporter plasmid resulted in dramatically reduced LUC signals compared with the control. This supports the hypothesis that WRKY75 is a direct transcriptional repressor of *GLK1* expression.

### ABA-induced leaf senescence delayed by SIBs is *WRKY75*-dependent

**Fig. 5.**
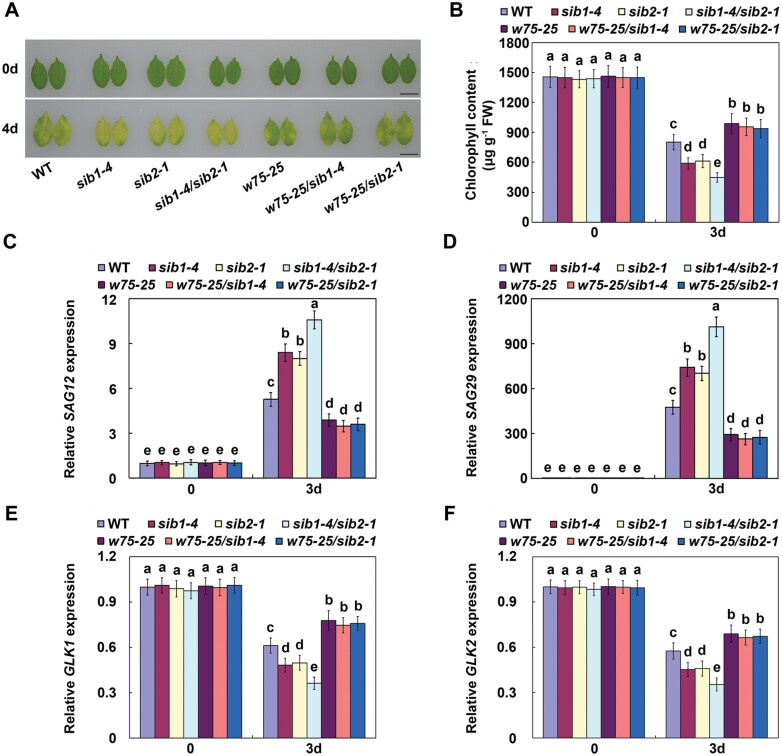
*SIB1/2* negatively modulate ABA-induced leaf senescence in a *WRKY75*-dependent manner. (A) Senescence phenotypes of the indicated genotypes treated with or without 100 µM ABA for 4 d. Scale bar =1 cm. (B) Chlorophyll content in the indicated genotypes treated with or without ABA for 3 d.(C-F) qRT–PCR analysis of *SAG12*, *SAG29*, *GLK1,* and *GLK2* transcript levels in the indicated genotypes treated with or without ABA for 3 d. Transcript levels of *SAG12*, *SAG29*,*GLK1* and *GLK2* in non-treated WT leaves were arbitrarily set to 1. *ACTIN2* and *UBQ5* were used as internal controls. For B-F, error bars represent ±SD from three independent biological replicates. Bars with different letters are significantly different from each other (ANOVA; *P*<0.05).

We have shown that SIBs interact with WRKY75, according to both Y2H and LCI assays (Fig. 2), and that SIBs and WRKY75 function in opposite ways in ABA-mediated leaf senescence and seed germination. We therefore deduced that the role of SIBs in ABA responses may be mediated by its interaction with WRKY75. To confirm our speculation, we examined whether the accelerated leaf senescence of *sib1* and *sib2* mutants is *WRKY75*-dependent. We then crossed both *sib1-4* and *sib2-1* mutants with *wrky75-25* to produce *wrky75-25/sib1-4* and *wrky75-25/sib2-1* (Rishmawi *et al.*, 2014). As shown in Fig. 5A, mutation of *SIB1* or *SIB2* could not accelerate ABA induced leaf senescence of *wrky75-25*. Consistent with the senescence phenotypes, the chlorophyll content and expression of both *SAG*s and *GLK*s were similar between *wrky75-25* and *wrky75-25/sib1-4* or *wrky75-25/sib2-1* (Fig. 5B–F). Thus, ABA-induced leaf senescence delayed by *SIB*s is *WRKY75*-dependent.

### SIB1 and SIB2 are negative interactors of WRKY75

**Fig. 6.**
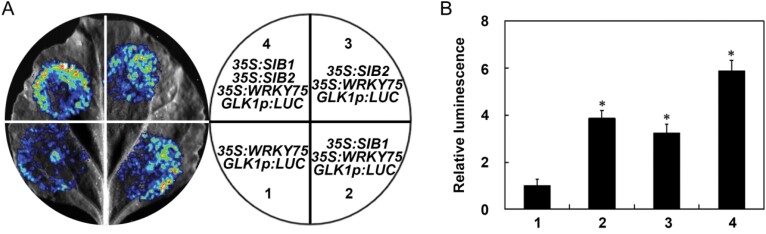
SIB1/2 repress the transcriptional activity of *WRKY75*. Transient transcriptional activity assays in *N. benthamiana* revealing the repression of *GLK1* by WRKY75. A representative leaf image is shown in (A), and the quantification of the corresponding relative luminescence intensities is shown in (B) by using *n*=15 independent leaves. Error bars represent ±SD.*P<0.05, Student’s *t*-test compared with control (*35S:WRKY75*+*GLK1p:LUC*,1).

Previous studies have revealed that VQ proteins themselves do not bind DNA directly but form complexes with several types of transcription factors to either promote or suppress their transcriptional activity, and finally exert their regulatory effects (Lai *et al.*, 2011; Li *et al.*, 2014; Jing and Lin, 2015; Lei *et al.*, 2017). Having demonstrated that SIB1 and SIB2 physically and genetically interact with WRKY75, we speculated that they might affect the transcriptional function of WRKY75. To test this possibility, we used the LUC reporter approach to analyze the effects of SIB1 and SIB2 on the transcriptional activity of WRKY75. *GLK1p:LUC* was again used as a reporter. Effector constructs were generated that contained either a *SIB1*, *SIB2*, or *WRKY75* gene driven by the CaMV*35S* promoter (*35S:SIB1*, *35S:SIB2*, and *35S:WRKY75*). As shown in Fig. 6, co-expression of *SIB1* or *SIB2* with *WRKY75* resulted in enhanced LUC signals compared with the expression of *WRKY75* alone (Fig. 6). More importantly, co-expression of *SIB1* and *SIB2* with *WRKY75* further enhanced the LUC signals compared with the expression of *WRKY75* alone, or co-expression of *WRKY75* and *SIB1* or *SIB2*. These results support the hypothesis that SIB1 and SIB2 act as negative interactors of WRKY75.

### WRKY75 promotes both ABA-mediated leaf senescence and seed germination in a *GLK*-dependent manner

**Fig. 7.**
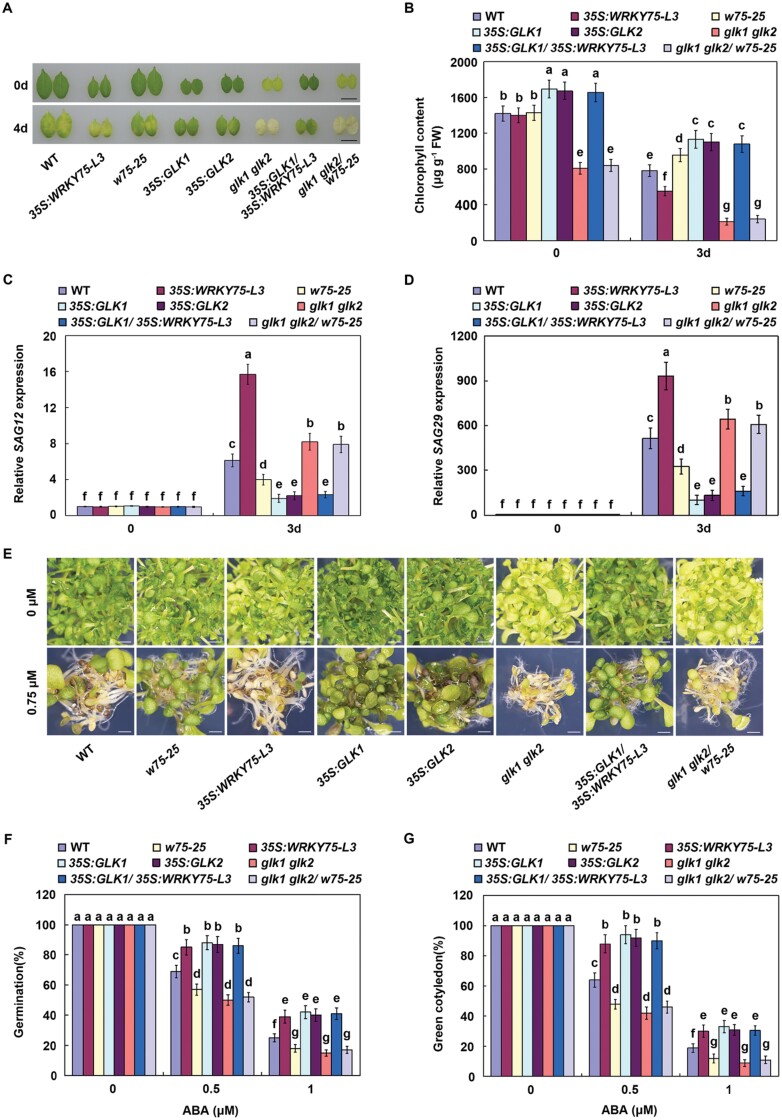
WRKY75 promotes both ABA-induced leaf senescence and seed germination in a *GLK*-dependent manner. (A) Senescence phenotypes of the indicated genotypes treated with or without 100 µM ABA for 4 d. Scale bar =1 cm. (B) Chlorophyll content in the indicated genotypes treated with or without 100 µM ABA for 3 d. (C, D) qRT–PCR analysis of *SAG12* and *SAG29* in the indicated genotypes treated with or without 100 µM ABA for 3 d. Transcript levels of *SAG12* or *SAG29* in non-treated WT leaves were arbitrarily set to 1. *ACTIN2* and *UBQ5* were used as internal controls. (E) Phenotypes of the indicated genotypes grown on half-strength MS medium with 0 or 0.5 µM ABA for 5 d. Scale bar =2 mm. (F) Germination rates of the indicated genotypes grown on half-strength MS medium with 0, 0.5, or 1 µM ABA for 3 d. (G) Cotyledon greening rates of the indicated genotypes grown on half-strength MS medium with 0, 0.5, or 1 µM ABA for 6 d. For B-D, F and G, error bars represent ±SD from three independent biological replicates. Bars with different letters are significantly different from each other (ANOVA; *P*<0.05).

The phenotypic analysis, and biochemical and molecular evidence indicated that WRKY75 positively modulates both ABA-mediated leaf senescence and seed germination through the direct inhibition of *GLK* expression. To further confirm this conclusion, the genetic relationship between *WRKY75* and *GLK*s was explored. The *wrky75-25* mutant was crossed with *glk1/glk2* double mutant to produce *glk1/glk2/wrky75-25*, and *35S:WRKY75-L3* was crossed with *35S:GLK1* to generate *35S:GLK1/35S:WRKY75-L3* (Supplementary Fig. S2). Then both the ABA-mediated leaf senescence and seed germination among these transgenic lines were examined. As shown in Fig. 7A, mutation of *WRKY75* could not delay leaf senescence of *glk1/glk2*, and overexpression of *WRKY75* also could not promote leaf senescence of *35S:GLK1*. Consistent with these senescence phenotypes, chlorophyll content and *SAG* expression were similar between *glk1/glk2* and *glk1/glk2/wrky75-25,* or between *35S:GLK1* and *35S:GLK1/35S:WRKY75-L3*(Fig. 7B–D). Similarly, mutation of *WRKY75* could not promote seed germination and early seedling growth of *glk1/glk2*, and overexpression of *WRKY75* also could not delay seed germination and early seedling growth of *35S:GLK1*(Fig. 7E–G). Thus, the genetic analysis indicated that *WRKY75* acts upstream of *GLK*s and functions as a positive regulator of both ABA-mediated leaf senescence and seed germination in a *GLK*-dependent manner.

## Discussion

Although VQ proteins have been identified in several plants, and substantial progress in their function has been obtained in recent years, our knowledge on their functions and mechanisms of action still remains largely unknown. Previous studies have demonstrated that VQ proteins constitute a class of transcription regulators that do not bind DNA directly but form complexes with transcription factors to fine-tune downstream gene transcription (Jing and Lin, 2015; Yuan *et al.*, 2021). Consequently, VQ proteins function as both positive and negative regulators to modulate plant growth and development, such as endosperm growth, seed size, and light morphogenesis, as well as stress responses, including plant immunity and several abiotic stresses (Yuan *et al.*, 2021). Here, we demonstrate that the VQ proteins SIB1 and SIB2 function as repressors of WRKY75 in ABA-mediated leaf senescence and seed germination.

We found that both *SIB1* and *SIB2* are expressed in senescing leaves and also induced by ABA; both *sib1* and *sib2* single mutants and their double mutants exhibit accelerated ABA-induced leaf senescence, while *SIB1*-overexpressing plants show dramatically delayed ABA-induced leaf senescence compared with WT plants (Fig. 1), indicating that both *SIB1* and *SIB2*act as negative regulators of ABA-mediated leaf senescence. These ABA-induced senescence phenotypes were correlated with the altered expression of several senescence-associated genes, such as *SAG12* and *SAG29* (Fig. 1). Similar to their role in ABA-mediated leaf senescence, *SIB*s also function redundantly and negatively in ABA-mediated seed germination. As the plant-specific protein family, the VQ family in Arabidopsis consists of 34 members with functional diversity (Yuan *et al.*, 2021). However, until now, there is little report about their involvement in the plant senescence response. Our results thus shed new insights into the importance of VQ proteins, especially in the plant senescence response.

As a class of transcription regulators, VQ proteins often interact with transcription factors to fine-tune the regulatory machinery associated with plant growth and development, and also response to diverse environmental stresses (Jing and Lin, 2015). Previous studies have provided evidence to show that VQ-WRKY interaction represents one important mechanism of action of the VQ proteins. Both SIB1 and SIB2 interact with WRKY33 to positively regulate plant defense against necrotrophic pathogens (Lai *et al.*, 2011). VQ20 can form complexes with both WRKY2 and WRKY34 to positively regulate pollen development (Lei *et al.*, 2017). In contrast, VQ9 physically interacts with WRKY8 to function antagonistically in the regulation of salt stress response (Hu *et al.*, 2013). Thus, VQ proteins can function as both activators and inhibitors of WRKY transcription factors, and they form complexes to fine-tune the complex regulatory networks. In this study, we also identified SIB1 and SIB2 as two interacting partners of WRKY75 using both *in vivo* and *in vitro* biochemical analyses. Expression analysis revealed that *WRKY75* is strongly induced by ABA at both mRNA and protein levels and is strongly expressed in senescing leaves, indicating that WRKY75 may also be involved in ABA-induced leaf senescence (Fig. 3). In contrast with *SIB*s, *wrky75* mutant plants show delayed ABA-induced leaf senescence, while *WRKY75*-overexpressing plants exhibit dramatically accelerated ABA-induced leaf senescence, when compared with the WT plants (Fig. 3). Similarly, WRKY75 also has a positive role in ABA-mediated seed germination(Fig. 3). Thus SIBs may form a complex with WRKY75 to modulate ABA-mediated leaf senescence and seed germination.

Plant senescence is precisely controlled by complex senescence-associated transcriptional regulatory networks, in which numerous transcription factors participate. Interestingly, WRKY proteins were identified as the second largest group of transcription factors in the Arabidopsis senescence transcriptome (Guo *et al.*, 2004), and accordingly, several WRKY members have been reported to participate in plant senescence regulation, including *WRKY6* (Robatzek and Somssich, 2002), *WRKY22* (Zhou *et al.*, 2011), *WRKY53* (Miao and Zentgraf, 2007), *WRKY54* (Besseau *et al.*, 2012), *WRKY57* (Jiang *et al.*, 2014), and *WRKY70* (Ulker *et al.*, 2007; Besseau *et al.*, 2012). Recently, WRKY75 was shown to participate in GA-mediated leaf senescence, and also participate in the formation of a tripartite amplification loop together with SA and reactive oxygen species to accelerate leaf senescence (Guo *et al.*, 2017; Zhang *et al.*, 2021). Despite their wide involvement in plant senescence, it is still unclear whether there are certain senescence-associated WRKY members that participate in leaf senescence through the ABA pathway. Here we identified WRKY75 as a critical component in ABA-induced leaf senescence.

Despite their functional diversity, WRKY proteins were found to perform their biological functions by directly binding to the W-box (TTGACC/T) present in their target promoters. Interestingly, we here revealed that WRKY75 can directly target *GLK*s through the W-boxes in their promoters, and subsequently regulate both ABA-mediated leaf senescence and seed germination (Fig. 4). Because of the contrasting expression pattern of both *WRKY75* and *GLK*s in *WRKY75* mutants and overexpression plants, and the reduced LUC signal in transient expression assays (Fig. 4), we deduced that WRKY75 functions as a negative regulator of *GLK*s. Genetic analysis further revealed that WRKY75 acts upstream of *GLK*s and participates in ABA-mediated leaf senescence and seed germination in a *GLK-*dependent manner (Fig. 7). Previously, WRKY75 was also demonstrated to directly activate several senescence-associated genes and *SA INDUCTIONDEFICIENT 2* (*SID2*), but repress *CATALASE2* (*CAT2*) during leaf senescence (Guo *et al.*, 2017; Zhang *et al.*, 2021). Thus, WRKY75 functions as an activator as well as a repressor to fine-tune leaf senescence.

Consistent with the opposite phenotypes in ABA-mediated leaf senescence and seed germination, we also observed opposite expression of *GLK*s between *SIB*s and *WRKY75* mutants or overexpression plants (Fig. 4). Furthermore, SIBs can repress the transcriptional inhibitory effect of WRKY75 on *GLK1* expression and also function by depending on *WRKY75* (Figs 5;6). SIBs thus may form a complex with WRKY75 and function to maintain the appropriate ABA signaling level, finally modulating ABA-associated responses. Previous studies have demonstrated that VQs interact with diverse transcription factors to affect their DNA-binding activity or transcriptional activation/inhibition ability. For example, SIBs enhanced the DNA-binding activity of WRKY33, while VQ9 reduced the DNA-binding activity of WRKY8 (Lai *et al.*, 2011; Hu *et al.*, 2013). Both VQ18 and VQ26 repressed the transcriptional activation ability of ABI5, while VQ20 promoted the transcriptional inhibition ability of WRKY2 and WRKY34 (Lei *et al.*, 2017; Pan *et al.*, 2018). Furthermore, SIBs function in *B. cinerea* defense responses in a *WRKY33*-dependent manner (Lai *et al.*, 2011). Thus, VQs perform their regulatory roles through interaction with diverse transcription factors, and function synergistically or antagonistically to fine-tune the complex regulatory networks.

**Fig. 8.**
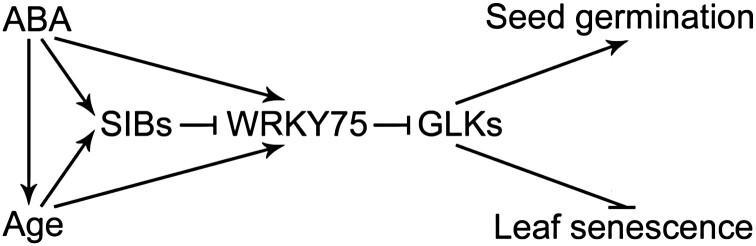
Model for SIB-WRKY75interaction in ABA-mediated leaf senescence and seed germination. Both *SIB*s and *WRKY75* are up-regulated during leaf senescence and induced by ABA. The ABA pathway generally promotes leaf senescence in Arabidopsis. SIBs interact with WRKY75 to inhibit its transcriptional function. *GLK*s negatively modulate ABA-mediated leaf senescence but positively modulate ABA-mediated seed germination. WRKY75 directly binds to the promoters of *GLK*s to repress their expression during ABA-mediated leaf senescence and seed germination.

Our results reveal the molecular mechanisms underlying the regulation of ABA-mediated leaf senescence and seed germination by the SIB-WRKY75 complex. They demonstrated that both SIBs and WRKY75 function as novel components of an ABA-mediated regulatory network, and they physically interact and function to fine-tune the ABA-mediated responses. Finally, a working model for the function of SIB-WRKY75 complex in ABA-mediated responses was proposed (Fig. 8). Both *SIB*s and *WRKY75* are up-regulated during leaf senescence and induced by ABA, and the proteins form a complex to regulate *GLK* expression during ABA-mediated leaf senescence and seed germination. Given that *SIB*s show slightly altered expression in ABA signaling mutants (e.g. *abi4* and *abi5;*Supplementary Fig. S3), *SIB1*-overexpressing plants still show ABA-promoted senescence (Fig. 1), and *wrky75* mutants and *GLK*-overexpressing plants also show ABA-dependent senescence (Fig. 2), we speculate that alternative ABA mechanisms may also participate in ABA-mediated leaf senescence. It will be interesting to further investigate how transcription and translation of *SIB*s or *WRKY75* are regulated upon ABA stimuli, and how they form protein complexes during this process. It will also be necessary to determine whether there exist other VQ members that participate in the senescence process. Further systematic analysis of the biological significance of VQ-WRKY interaction will add to our understanding of the involvement of VQ-WRKY complexes in diverse biological processes.

## Supplementary data

The following supplementary data are available at *JXB* online.

Fig. S1. Confirmation of *SIB1* overexpressing plants.

Fig. S2. Confirmation of *GLK1*, *GLK2*, and *WRKY75* overexpressing plants.

Fig. S3.Identification of *SIB*s in ABA synthesis or signaling mutants.

Table S1. Primers used in this study.

erab391_suppl_Supplementary_Figures_S1-S3_Table_S1

## Data Availability

Sequence data from this article can be found in the GenBank/EMBL libraries (https://www.ncbi.nlm.nih.gov/) under the following accession numbers: *SIB1* (At3G56710), *SIB2* (At2G41180), *WRKY75* (AT5G13080), *GLK1* (AT2G20570), *GLK2* (AT5G44190), *SAG12* (AT5G45890), *SAG29* (AT5G13170), *ACTIN2* (AT3G18780), and *UBQ5* (AT3G62250).
