# Supplementary material for: Arabidopsis SIGMA FACTOR BINDING PROTEIN1 (SIB1) and SIB2 inhibit WRKY75 function in abscisic acid-mediated leaf senescence and seed germination
Source: J Exp Bot. 2021 Aug 26;73(1):182–96. doi: 10.1093/jxb/erab391 (PMC8730687; doi:10.1093/jxb/erab391)
Supplement: erab391_suppl_Supplementary_Figures_S1-S3_Table_S1 [file erab391_suppl_supplementary_figures_s1-s3_table_s1.pdf]

Supplementary data:

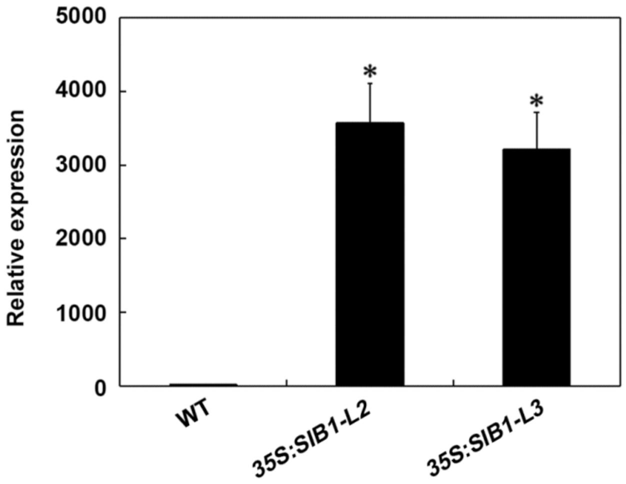

Fig. S1. Determination of *SIB1* overexpressing plants. qRT-PCR analysis for *SIB1*-overexpressing plants. Transcript levels of *SIB1* in WT were arbitrarily set to 1. *ACTIN2* and *UBQ5* were used as internal controls. Values are means ± SD of three independent biological replicates. * P < 0.05, Student's t-test compared with WT.

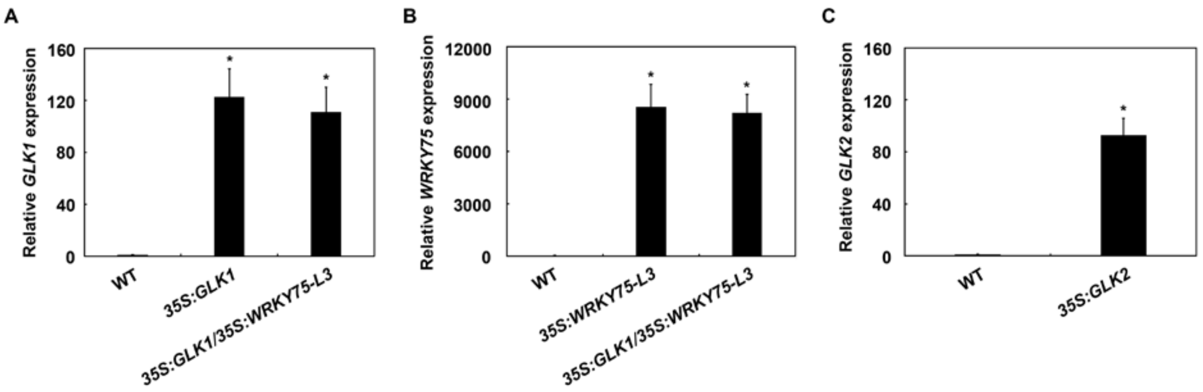

Fig. S2. Determination of *GLK1*, *GLK2* and *WRKY75* overexpressing plants. qRT-PCR analysis for *GLK1*-、*GLK2*- and *GLK1/WRKY75*-overexpressing plants. Transcript levels of *GLK1*、*GLK2* or *WRKY75* in WT were arbitrarily set to 1. *ACTIN2* and *UBQ5* were used as internal controls. Values are means ± SD of three independent biological replicates. * P < 0.05, Student's t-test compared with WT.

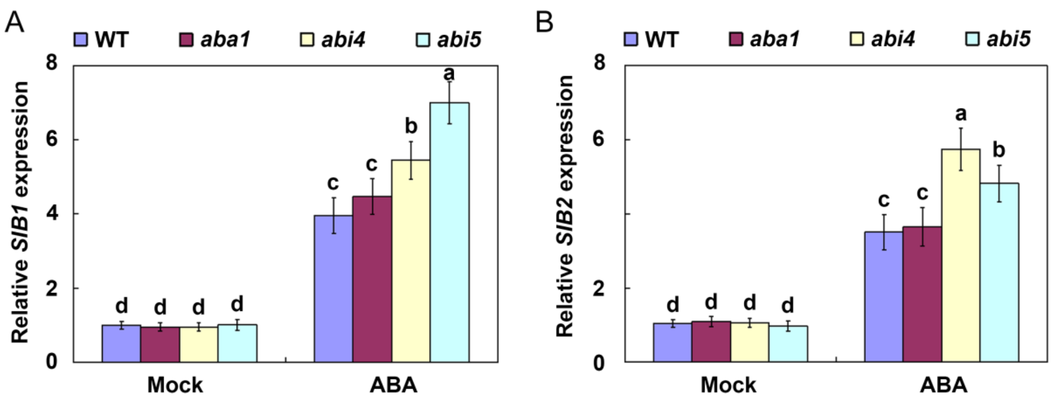

Fig. S3. Determination of *SIBs* expression in ABA synthesis or signaling mutants. qRT-PCR analysis of *SIBs* in the indicated genotypes. Transcript levels of *SIB1* and *SIB2* in non-treated WT leaves were arbitrarily set to 1. *ACTIN2* and *UBQ5* were used as internal controls. Error bars represent ±SD from three independent biological replicates. Bars with different letters are significantly different from each other (ANOVA; $P < 0.05$).

Supplementary Table S1.

Primers used to qRT-PCR analysis of gene expression:

GLK1-q1:	CCAAACACTTACCTCCGCCT
GLK1-q2:	GGCGGTGCTCTAAATCTCGT
GLK2-q1:	TTCAAGCATCGGTGTTCCCA
GLK2-q2:	TTCAGTCCCAAAGGAAGCGG
SAG12-q1:	ATCCAAAAGCAACTTCTATTACAGG
SAG12-q2:	CCACTGCCTTCATCAGTGC
SAG29-q1:	GCCACCAGGGAGAAAAGG
SAG29-q2:	CCACGAAATGTGTTACCATTAGAA
SIB1-q1:	CGACTTTTCTCACCACGACA
SIB1-q2:	TCGGAGGAAGGGGAATAGAT
SIB2-q1:	GTCAAGACGCCGCTGATCTA
SIB2-q2:	GCCGACATCTGAGGAGCATT
WRKY75-q1:	ATATGGCCAAAAGGCCGTCA
WRKY75-q2:	TGCTCGAAGTTTTCGGTGGA
ACTIN2-q1:	TGTGCCAATCTACGAGGGTTT
ACTIN2-q2:	TTTCCCGCTCTGCTGTTGT
UBQ5-q1:	GTTAAGCTCGCTGTTCTTCAGT
UBQ5-q2:	TCAAGCTTCAACTCCTTCTTTC

Primers used to LCI analysis:

cLUC-SIB1-1:	ATCCCGGGGCATGGAGTCATCATCGTCGACTTTT
cLUC-SIB1-2:	ATCCCGGGTTCACATAGAATCGATGCTTCCAAA
cLUC-SIB2-1:	ATCCCGGGGCATGGATCAGTCATCATCAACGTTG
cLUC-SIB2-2:	ATCCCGGGTTCAGAGAGAACCAATGCTTCCTAAA
nLUC-WRKY75-1:	ATCCCGGGGCATGGAGGGATATGATAATGGGTC
nLUC-WRKY75-2:	ATCCCGGGTGAAAGAAGAGTAGATTTGCATTTGAG

Primers used to yeast two hybrid:

AD-SIB1-1:	ATAGAATTCATGGAGTCATCATCGTCGACTTTT
AD-SIB1-2:	ATAGGATCCATTATCACATAGAATCGATGCTTCCA
AD-SIB2-1:	ATAGAATTCATGGATCAGTCATCATCAACGTT
AD-SIB2-2:	ATAGAGCTCTCAGTGCCGAAACAAAACATT
BD-WRKY75-1:	AAGAATTCATGGAGGGATATGATAATGGG
BD-WRKY75-2:	ATAGGATCCCTAGAAAGAAGAGTAGATTTGCATT

Primers used to transactivation analysis:

GLK1p-LUC-1:	ATCCCGGGTAGCTATTTCCAAGTTAACAGGAGAATC
GLK1p-LUC-2:	ATCCCGGGCGATCAATCTTCACTTGTAGATCCAA
35S-SIB1-1:	ATGGATCCATGGAGTCATCATCGTCGACTTTT
35S-SIB1-2:	ATTCTAGATCACATAGAATCGATGCTTCCAAA
35S-SIB2-1:	ATTCTAGAATGGATCAGTCATCATCAACGTTG

35S-SIB2-2: ATGTCGACTCAGAGAGAACCAATGCTTCCTAAA
35S-WRKY75-1: ATGGATCCATGGAGGGATATGATAATGGGTC
35S-WRKY75-2: ATGTCGACCTAGAAAGAAGAGTAGATTTGCATTTG

Primers used to mutant identification:
WRKY75-m1: TTGGAGATTGATTCGAATTGG
WRKY75-m2: AACACGTACACGACGTCATTG
WRKY75-m3: TCGAGCATATTCTCACTCAAATGC
WRKY75-m4: GGATCCAAAGATTTCAGGCTCAATTAC
Spm32: TACGAATAAGAGCGTCCATTTTAGAGTGA
Salk-KO: AAACGTCCGCAATGTGTTAT
sib1-4-LP: CGATGAGAACTCGATAACCTGAC
sib1-4-RP: AATCATCCCACCTATCGGAAG
sib2-1-LP: GCGTCTATTATACAGAGATGGGG
sib2-1-RP: TGTCATGGAGACACGATGAAC
sm_KO: TACGAATAAGAGCGTCCATTTTAGAGTGA

Primers used to generate SIB1 overexpression transgenic plants:
35S-SIB1-GFP-1: ATGGATCCATGGAGTCATCATCGTCGACTTT
35S-SIB1-GFP-2: ATTCTAGACATAGAATCGATGCTTCCAAAGTC

Primers used to Pull-Down:
GST-SIB1-1: ATGGATCCATGGAGTCATCATCGTCGACTTT
GST-SIB1-2: ATGAATTCCATAGAATCGATGCTTCCAAAGTC
GST-SIB2-1: ATGAATTCATGGATCAGTCATCATCAACGTTG
GST-SIB2-2: ATCTCGAGGAGAGAACCAATGCTTCCTAAAGC
His-WRKY75-1: ATGGATCCATGGAGGGATATGATAATGGGTC
His-WRKY75-2: ATGAATTCGAAAGAAGAGTAGATTTGCATTTGAG

Primers used to qRT-PCR amplification of *GLK1* or*GLK2* promoter:
GLK1-W1-1: CAAAACCACTCAAATATTCAACT
GLK1-W1-2: AATTCCACATACTTTCAGGCC
GLK1-W2,3-1: CTTCCACAGCTTAATTTGCGAG
GLK1-W2,3-2: CTTGGTTTTGGTCTGTCCCAAC
GLK1-W4-1: CCACATTTAGAACTAGGCATAT
GLK1-W4-2: GATACATGTAAACATCTGATCGTTTG
GLK2-W1-1: GAGGCACCAATTTTTTATGCCAC
GLK2-W1-2: GTCTCTATAACACATGTTATTGAGTC
GLK2-W2,3,4-1: ACGCATTTGCTTAATTGCTAGTCG
GLK2-W2,3,4-2: GACAGTTTCCTTTTAAACATTATGG
